# The Complete Mitochondrial Genome of the Land Snail *Cornu aspersum* (Helicidae: Mollusca): Intra-Specific Divergence of Protein-Coding Genes and Phylogenetic Considerations within Euthyneura

**DOI:** 10.1371/journal.pone.0067299

**Published:** 2013-06-24

**Authors:** Juan Diego Gaitán-Espitia, Roberto F. Nespolo, Juan C. Opazo

**Affiliations:** 1 Instituto de Ciencias Ambientales y Evolutivas, Universidad Austral de Chile, Valdivia, Chile; 2 Programa de Doctorado en Ciencias mención Ecología y Evolución, Facultad de Ciencias, Universidad Austral de Chile, Valdivia, Chile; Wuhan Botanical Garden, Chinese Academy of Sciences, China

## Abstract

The complete sequences of three mitochondrial genomes from the land snail *Cornu aspersum* were determined. The mitogenome has a length of 14050 bp, and it encodes 13 protein-coding genes, 22 transfer RNA genes and two ribosomal RNA genes. It also includes nine small intergene spacers, and a large AT-rich intergenic spacer. The intra-specific divergence analysis revealed that *COX1* has the lower genetic differentiation, while the most divergent genes were *NADH1*, *NADH3* and *NADH4*. With the exception of *Euhadra herklotsi*, the structural comparisons showed the same gene order within the family Helicidae, and nearly identical gene organization to that found in order Pulmonata. Phylogenetic reconstruction recovered Basommatophora as polyphyletic group, whereas Eupulmonata and Pulmonata as paraphyletic groups. Bayesian and Maximum Likelihood analyses showed that *C. aspersum* is a close relative of *Cepaea nemoralis*, and with the other Helicidae species form a sister group of *Albinaria caerulea*, supporting the monophyly of the Stylommatophora clade.

## Introduction

Mitochondria, powerhouses of the cell, are in charge of producing energy in the form of Adenosine triphosphate (ATP) that is usable by the cell in eukaryotic organisms. The metabolic pathway where this occurs is the oxidative phosphorylation (OXPHOS), and it requires a whole system of protein complexes, the electron transport chain (ETC), that are anchored to the inner membrane of the mitochondria. The enzymes that belong to the ETC are encoded by both mitochondrial (mtDNA) and nuclear (nDNA) genomes [1], where the nDNA-encoded peptides have mostly structural functions, whereas mtDNA-encoded peptides constitute the main catalytic centres [2].

**Figure 1:**
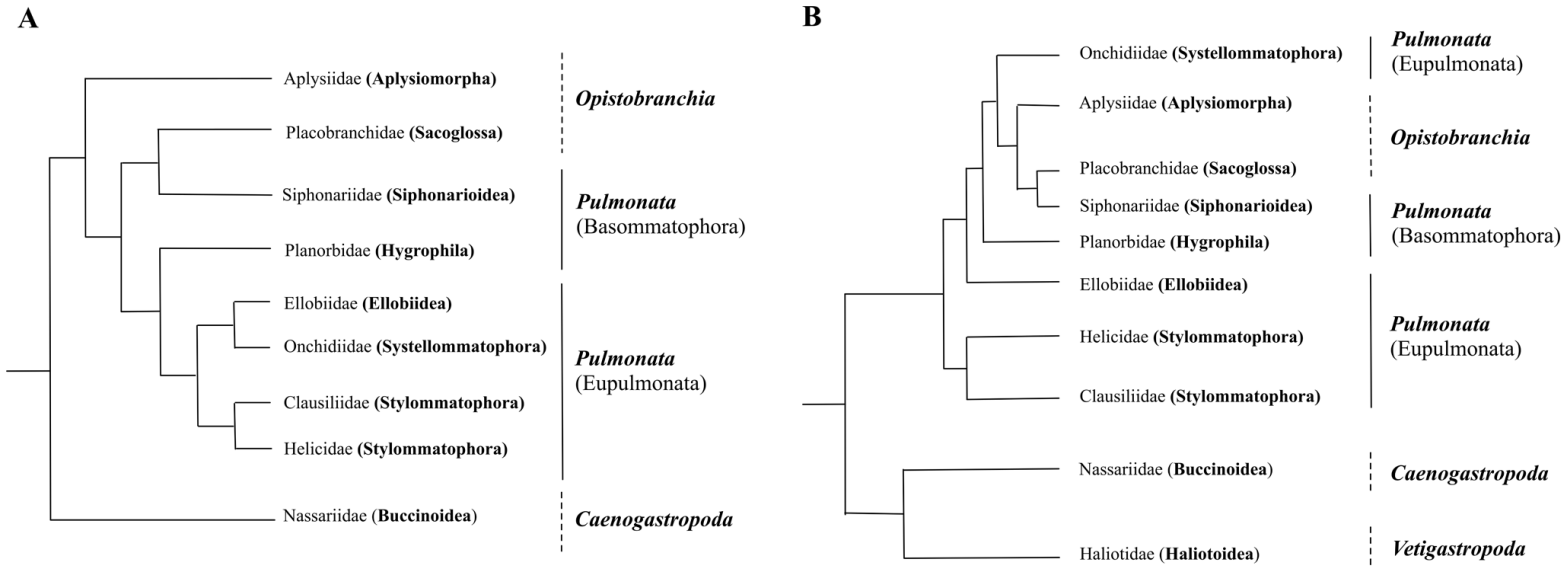
Competing hypotheses regarding the phylogenetic relationships among Euthyneuran species. A) The monophyly of Eupulmonata has been documented based on the combination of mitochondrial and nuclear genes [13], [17], [18], and B) The paraphyly of Eupulmonata has been recovered using complete mitochondrial genomes [4], [16].

The mitochondrial genome of metazoans is typically a circular double stranded DNA molecule of about 12–20 kb length, which contains 37 genes including 13 protein-coding genes, 2 ribosomal RNAs (*12S rRNA* and *16S rRNA*) genes, and 22 transfer RNAs (tRNAs) [3]. Additionally it has an AT-rich non-coding region that contains the potential origin for mitochondrial DNA replication (POR) [4] and RNA transcription (i.e., the mitochondrial control region) [3], [5]. Over the last decades, mitochondrial genomes have been used for a wide range of comparative studies as phylogenetic markers to resolve evolutionary relationships [6]. Particularly the phylum mollusca, especially the Euthyneura clade, has been subject of intense debate regarding their phylogenetic relationships [4], [6]–[13]. The lineages associated with this group (i.e., Opistobranchia and Pulmonata) have been the subject of long controversy due to the different phylogenetic results obtained with morphological [14], [15] and molecular data [4], [11], [16]. In particular, within Pulmonata some authors had found contradictory results from molecular phylogenetic reconstructions. For example, the monophyly of Eupulmonata (Fig. 1A) has been documented based on the combination of mitochondrial and nuclear genes [13], [17], [18], whereas the paraphyly of this group (Fig. 1B) has been recovered using complete mitochondrial genomes [4], [16].

Although a great number of mitochondrial genomes is reported in the Organelle Genome Resources of the NCBI for Euthyneura, there are some clades such as Stylommatophora that have few representatives, in which some of the mitochondrial genomes has been criticised because of the poor quality of its sequence [4]. In principle, the addition of new mitochondrial genomes from species of the family Helicidae will increase the taxonomic sampling in a clade that today has few representatives (Stylommatophora), and from a phylogenetic standpoint, will add valuable information to understand evolutionary relationships at different taxonomic levels. Moreover this information will also help to elucidate genomic features, such as gene order, that are unique to this group of snails, and those that are shared with other clades.

Here we report the mitochondrial genome of three individuals of the land snail *Cornu aspersum*, one of the most widespread invasive species in the world [19], [20]. This species is native from the West Mediterranean, and since the Holocene has successfully colonized all continents except Antarctica [21]. Currently, its distribution extends to vast areas in the temperate and subtropical regions of the world where it is found in agricultural areas, parks and domestic gardens within cities. At these locations, *C. aspersum* snails are quite abundant thus contributing to its consideration as an important pest [22]–[24]. The mitochondrial genomes of *C. aspersum* reported in this work correspond to three individuals from three populations in a latitudinal gradient from northern to southern Chile, characterized by contrasting climatic conditions [25]. These populations are genetically differentiated [26] and have been subject of at least three different events of introduction during the last century [27]. Such factors could underlie the physiological differences exhibited by these populations [25], [28], which are probably evidenced by the divergence of those genes related to the OXPHOS and ETC. Results from our comparative genomic analysis revealed that the mitochondrial genome of *C. aspersum* encodes for the 37 genes typical of most metazoans, and is characterized by the highly conserved gene order and gene content of pulmonate gastropods, with only few gene rearrangements that are shared within Helicidae. In accordance to previous studies, where complete mitochondrial genomes were used to infer phylogenetic relationships, we recovered Basommatophora as polyphyletic group, whereas Eupulmonata and Pulmonata as paraphyletic groups.

## Materials and Methods

### Specimen Collection and DNA Isolation

Adult individuals of *Cornu aspersum* were collected from three populations in Chile across a latitudinal range of approximately 1300 km: La Serena (29°54′ S, 71°15′ W), Constitución (35°20′ S, 72°25′ W) and Valdivia (39° 38′ S, 73° 5′ W). We selected these three localities based on their significant genetic [27] and climatic differentiations [25]. Snails were transported to the laboratory, and maintained at 20°C with a fixed photoperiod of 14L:10D. Additionally, snails were kept in humid litter soil and water was given *ad libitum* until DNA extractions were performed. DNA from a single snail of each population was obtained by the isolation of intact mitochondria from approximately 120 mg of fresh foot tissue, using the Mitochondrial Isolation Kit for Tissue (Thermo Scientific). The isolated mitochondrial pellet of each snail was used for the mtDNA extraction using the Mitochondrial DNA Isolation kit (BioVision).

### Mitochondrial Genomes Sequencing and Assembly

The shotgun libraries of *C. aspersum* were sequenced using a combination of 454 (Roche Genome Sequencer GS FLX Titanium) and Sanger sequencing technologies on ABI 3730XL sequencers by Eurofins MWG Operon (Huntsville, USA). DNA samples were nebulized, individually bar-coded to perform emulsion-based clonal ampiflication (emPCR) and sequenced to approximately 20-fold coverage. After sequencing, raw sequence files were proof read, separated, and assembled, according to the bar-codes, into contigs in Celera Assembler v.6.1 [29]. Assembly data was evaluated with the statistical overview and quality scoring files of each single read. Sequences obtained in this work were deposited in GenBank under the accession numbers JQ417194 (La Serena), JQ417195 (Constitución) and JQ417196 (Valdivia).

### Genome Annotation

Fragments of the whole mitochondrial DNA sequence were analyzed in MacClade 4.08 [30] and MEGA v.5.1 [31]. To control for sequencing errors, each partial sequence was evaluated at least twice. Ambiguous base pairs were resolved manually according to Roche’s flowcharts and the corresponding quality score values for each base in the reads. Annotations and editing procedures of the mitochondrial genomes of *C. aspersum* were done in Geneious v 4.8.5 [32]. Mitochondrial genes were identified by sequence comparison using DOGMA [33] and BLAST searches at NCBI (with the BlastN and BlastX algorithms [34]) against other Eupulmonata sequences (Table 1). The limits of both protein coding and ribosomal RNA genes were adjusted manually based on location of adjacent genes, and the presence of start and stop codons. Transfer RNA genes were located using ARWEN v.1.2 [35], DOGMA [33] and tRNAscan-SE v.1.21 [36], by means of the generalized invertebrate mitochondrial tRNA settings, after which they were manually adjusted based on specific anticodons in regions between identified genes.

**Table 1:** List of the Euthyneuran species included in the present study.

Taxon	Order	Family	Species name	GenBank
PULMONATA				
*Eupulmonata*	Stylommatophora			
		Helicidae	*Cornu aspersum*	JQ417194
			*Cornu aspersum*	JQ417195
			*Cornu aspersum*	JQ417196
			*Cepaea nemoralis*	CMU23045
			*Cylindrus obtusus*	JN107636
			*Euhadra herklotsi*	Z71693 - Z71701
		Clausiliidae	*Albinaria caerulea*	X83390
		Succineidae	*Succinea putris*	JN627206
	Systellommatophora			
		Onchidiidae	*Onchidella celtica*	AY345048
			*Onchidella borealis*	DQ991936
			*Platevindex mortoni*	GU475132
			*Peronia peronii*	JN619346
	Trimusculoidea			
		Trimusculidae	*Trimusculus reticulatus*	JN632509
	Amphibolidea			
		Amphibolidae	*Salinator rhamphidia*	JN620539
	Ellobiidea			
		Ellobiidae	*Myosotella myosotis*	AY345053
			*Auriculinella bidentata*	JN606066
			*Ovatella vulcani*	JN615139
			*Pedipes pedipes*	JN615140
*Basommatophora*	Hygrophila			
		Planorbidae	*Biomphalaria glabrata*	AY380531
		Lymnaeidae	*Radix balthica*	HQ330989
			*Galba pervia*	JN564796
	Siphonarioidea			
		Siphonariidae	*Siphonaria pectinata*	AY345049
			*Siphonaria gigas*	JN627205
**OPISTHOBRANCHIA**				
	Aplysiomorpha			
		Aplysiidae	*Aplysia californica*	AY569552
			*Aplysia dactylomela*	DQ991927
	Sacoglossa			
		Placobranchidae	*Elysia chlorotica*	EU599581
		Volvatellidae	*Ascobulla fragilis*	AY345022
	Cephalaspidea			
		Hydatinidae	*Hydatina physis*	DQ991932
		Acteonidae	*Pupa strigosa*	AB028237
	Notaspidea			
		Pleurobranchidae	*Berthellina ilisima*	DQ991929
	Nudibranchia			
		Chromodorididae	*Chromodoris magnifica*	DQ991931
		Polyceridae	*Roboastra europaea*	AY083457
**BASAL HETEROBRANCHIA**				
	Pyramidelloidea			
		Pyramidellidae	*Pyramidella dolabrata*	AY345054
**CAENOGASTROPODA**				
*Neogastropoda*	Buccinoidea			
		Nassariidae	*Ilyanassa obsoleta*	DQ238598
**VETIGASTROPODA**				
	Haliotoidea			
		Haliotidae	*Haliotis rubra*	AY588938

### Alignment and Divergence

Nucleotide translated sequences for the protein-coding genes from the three mtDNA sequences of *C. aspersum,* and other Euthyneuran species (Table 1) were aligned using the L-INS-I strategy from MAFFT [37]. Nucleotide alignments were generated using the amino acid alignment as a template using the web-based program TranslatorX [38]. Our taxonomic sampling included representative species of Pulmonata, Opistobranchia, Caenogastropoda and Vetigastropoda clades (Table 1). The last two groups were used as outgroups. The nucleotide and amino acid composition were estimated by Geneious v 4.8.5 [32]. Individual alignments were concatenated prior to phylogenetic analysis. Additionally, intra-specific divergences for each protein-coding gene were calculated based on these alignments. A p-distance method was performed using 1000 bootstrap replications for variance estimation using the program MEGA v.5.1 [31].

### Phylogenetic Analyses

Best Partition Scheme (BPS) analyses for the concatenated alignments were conducted with the program PartitionFinder [39], using the Bayesian Information Criterion (BIC) and a heuristic search algorithm. A total of 39 data blocks were defined, following the criteria of one data block for each codon position in each gene. Maximum Likelihood (ML) inference was performed using the graphical interface version (RAxML-GUI, [40]) of RAxML v.7.2.6 software [41]. Maximum likelihood trees were estimate under the GTRGAMMA substitution model and node support was calculated via rapid bootstrapping analyses of 1000 pseudo-replicates. In addition, Bayesian analyses were conducted using MrBayes v.3.2 [42]. Two simultaneous independent runs were performed for 10,000,000 iterations of a Markov Chain Monte Carlo algorithm, with six simultaneous chains, sampling every 1000 generations. The rate parameter was allowed to vary, and the parameter estimation was “unlinked” for the following parameters: the shape of the gamma distribution, the substitution matrix, the proportion of invariable sites, and the estimation of state frequencies. The “temperature” parameter was set at 0.2. Support for the nodes and parameter estimates were derived from a majority rule consensus of the last 5,000 trees sampled after convergence. The average standard deviation of split frequency remained <0.01 after the burn-in threshold.

### Ethics Statement

This study did not involve endangered or protected species and was carried out in strict accordance with the recommendations in the Guide for the Care and Use of Laboratory Animals of the Comisión Nacional de Investigación Científica y Tecnológica de Chile (CONICYT). All experiments were conducted according to current Chilean law. The protocol was approved by the Committee on the Ethics of Animal Experiments of the Universidad Austral de Chile (Permit Number: 02-2011). Because snails were obtained from public parks and gardens, no specific permissions were required for any of the three locations involved in this study (La Serena, Constitución and Valdivia).

## Results

### Genome Structural Features

**Figure 2:**
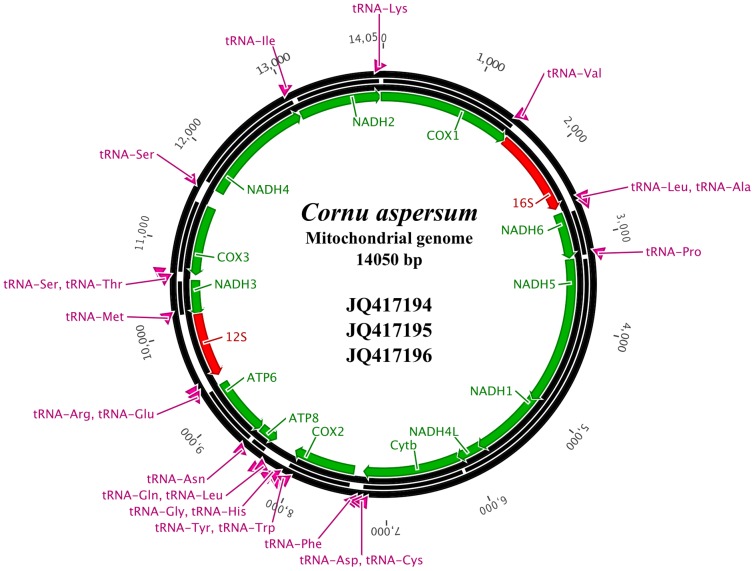
Schematic representation of the mitochondrial genome of *Cornu aspersum*. The molecule contains 13 protein-coding genes, two rRNAs and 22 tRNAs. Arrows indicate the transcription direction.

The size of the mitochondrial genome of *C. aspersum* is 14050 bp (Fig. 2), and contains 13 protein-coding genes, 22 transfer RNA genes, and two ribosomal RNA genes (Fig. 2). From these 37 genes, 13 are coded on the minus strand: *tRNA-Q*, *tRNA-L2*, *ATP8*, *tRNA-N*, *ATP6*, *tRNA-R*, *tRNA-E*, *12S-RNA*, *tRNA-M*, *NADH3*, *tRNA-S1*, *tRNA-T*, and *COX3* (Fig. 2). The mitochondrial genome of *C. aspersum* also includes nine small size-variable intergene spacer regions ranging from 2 to 47 bp, and a large AT-rich (81.2%) intergenic region (186 bp) located between *COX3* and *tRNA-S2* (i.e., Second Serine). This large intergene spacer contains three tandem repeated sequences (ATTATTA, ATTAGTG and TATAAATAT) of 7 bp length, distributed across the non-coding region. There are small gene overlaps at 14 gene borders, the largest has a length of 10 nucleotides and is located between *NADH3* and *tRNA-S1* (i.e., First Serine). The overall base composition of these mitochondrial genomes shows a high AT content (69.9%; Table 2).

**Table 2:** Structural features of the Eupulmonata mitochondrial genomes.

	Helicidae				Clausiliidae	Succineidae	Onchidiidae				Trimusculidae	Amphibolidae	Ellobiidea			
	*Cornu aspersum*	*Cepaea nemoralis*	*Cylindrus obtusus*	*Euhadra herklotsi*	*Albinaria caerulea*	*Succinea putris*	*Onchidella borealis*	*Onchidella celtica*	*Platevindex mortoni*	*Peronia peronii*	*Trimusculus reticulatus*	*Salinator rhamphidia*	*Myosotella myosotis*	*Auriculinella bidentata*	*Ovatella vulcani*	*Pedipes pedipes*
Total size	14050	14100	14610	14500	14130	14092	14510	14150	13991	13968	14044	14007	14246	14135	14274	16708
%A	30.8	26.2	25.8	29.5	32.8	33.8	29.3	25.3	27.3	27.1	26.4	26.7	23.7	25.8	25	28.6
%T	39.1	33.6	35.8	39.9	37.9	42.9	37	34.1	35.7	37.3	34.7	35.6	31.3	30.9	29.7	33.7
%C	13.6	18.9	16.6	14.1	13.8	10.8	18	18.9	26.8	15.4	18.2	16.9	21.3	20.4	21.6	18.4
%G	16.5	21.3	21.9	16.4	15.5	12.1	15.7	21.8	20.2	20.3	20.6	20.8	23.6	22.6	23.7	19.3
%A+T	69.9	59.8	61.5	69.4	70.7	76.7	66.3	59.4	63	64.4	61.1	62.3	55	56.7	54.7	62.3
%G+C	30.1	40.2	38.5	30.6	29.3	23.3	33.7	40.7	37	35.6	38.9	37.7	45	43.3	45.3	37.7
POR	186	158	189	43	42	47	41	43	23	54	47	44	46	44	44	397
12S rRNA	708	710	714	697	759	755	704	708	695	714	719	714	712	704	711	786
16S rRNA	984	1215	938	1024	1035	1020	1289	1056	1042	1033	1057	1025	1089	1047	1057	1138
Cox1	1530 (TTG/TAA)	1492 (TTG/TAA)	1527 (TTG/TAA)	1445 (TTG/T )	1529 (TTG/TA)	1548 (TTG/TAG)	1527 (TTG/TAA)	1527 (TTG/TAG)	1494 (ATT/TAA)	1525 (TTG/T)	1527 (TTG/TAG)	1527 (TTG/TAA)	1527 (ATG/TAA)	1533 (TTG/TAA)	1533 (TTG/TAA)	1527 (TTG/TAA)
Cox2	664 (ATG/T)	654 (ATT/TAG)	687 (ATG/TAG)	684 (TTG/TAG)	685 (ATG/TAA)	649 (ATG/T)	672 (TTG/TAA)	681 (TTG/TAA)	666 (ATG/TAA)	666 (TTG/TAA)	666 (TTG/TAA)	664 (GTG/T)	669 (GTG/TAA)	687 (TTG/TAG)	666 (TTG/TAG)	681 (GTG/TAA)
Cox3	780 (ATG/TAA)	814 (ATA/T)	813 (ATG/TAA)	644 (ATG/T)*	780 (ATG/TAA)	783 (ATG/TAG)	778 (ATG/T)	778 (ATG/T)	810 (ATA/TAA)	804 (ATG/T)	778 (ATG/T)	781 (ATG/T)	778 (ATG/T)	778 (ATG/T)	778 (ATG/T)	780 (GTG/TAG)
Cytb	1099 (ATA/T)	1143 (ATA/T)	1126 (ATG/T)	1035 (GTA/T)*	1103 (ATA/TA)	1107 (TTG/TAG)	1108 (TTG/T)	1122 (ATT/TAA)	1099 (ATT/T)	1108 (TTG/T)	1110 (TTG/TAA)	1111 (TTG/T)	1110 (TTG/TAG)	1111 (TTG/T)	1110 (TTG/TAA)	1108 (TTG/T)
Nadh1	873 (ATG/TAG)	883 (ATA/T)	873 (ATA/TAG)	891 (ATT/TAA)	900 (ATG/TAA)	916 (TTG/T)	906 (TTG/TAA)	906 (TTG/TAA)	882 (ATT/TAA)	906 (TTG/TAA)	906 (TTG/TAA)	958 (TTG/T)	882 (ATT/TAG)	906 (GTG/TAA)	906 (TTG/TAA)	903 (ATG/TAG)
Nadh2	927 (ATA/TAG)	946 (ATG/T)	979 (ATA/T)	928 (ATG/T)	924 (ATG/TAA)	975 (TTG/TAA)	922 (GTG/TAA)	922 (ATG/T)	922 (GTG/T)	939 (GTG/TAG)	916 (TTG/T)	925 (TTG/T)	948 (ATG/TAG)	945 (ATG/TAG)	942 (CTG/T)	927 (ATG/TAA)
Nadh3	343 (ATG/T)	405 (ATG/T)	357 (ATA/TAA)	184 (TTA/T)*	352 (ATA/T)	352 (ATG/T)	352 (ATG/TAA)	352 (ATG/T)	279 (TTG/TAA)	327 (ATT/TAA)	357 (ATG/TAG)	349 (TTG/T)	334 (ATA/T)	354 (TTG/TAA)	354 (TTG/TAA)	372 (ATG/TAA)
Nadh4	1293 (ATG/TAA)	1252 (ATA/T)	1329 (ATC/TAA)	975 (TTG/T)*	1314 (ATG/TAA)	1326 (ATG/TAA)	1305 (ATA/TAA)	1308 (GTG/TAA)	1326 (ATG/TAA)	1318 (TTG/T)	1306 (TTG/T)	1311 (TTG/TAG)	1305 (TTG/TAA)	1311 (TTG/TAG)	1308 (TTG/TAG)	1302 (ATG/TAG)
Nadh4L	264 (ATA/TAG)	238 (ATA/T)	250 (ATA/T)	280 (ATG/T)	298 (ATG/T)	275 (ATA/TA)	274 (ATG/TAA)	268 (ATG/T)	288 (ATG/TAA)	283 (ATG/T)	286 (ATG/T)	279 (TTG/TAG)	291 (TTG/TAA)	327 (TTG/TAG)	286 (TTG/T)	288 (GTG/TAG)
Nadh5	1677 (ATA/TAA)	1686 (ATG/TAG)	1680 (TTG/TAG)	846 (ATA/T)*	1638 (ATT/TAG)	1680 (ATG/TAA)	1612 (ATC/TAA)	1641 (GTG/TAG)	1536 (ATG/TAG)	1671 (TTG/TAG)	1680 (TTG/TAG)	1671 (TTG/TAG)	1656 (GTG/TAG)	1665 (ATA/TAG)	1671 (TTG/T)	1701 (ATG/TAG)
Nadh6	489 (TTG/TAA)	493 (ATT/TA)	498 (ATA/TAG)	141 (ATG/T)*	468 (ATG/TAA)	453 (ATG/TAA)	459 (ATG/TAA)	465 (TTG/TAA)	468 (ATT/TAA)	468 (ATT/TAA)	456 (TTG/TAG)	474 (TTG/TAA)	468 (ATA/TAG)	483 (ATT/TAA)	480 (ATT/TAG)	462 (TTG/TAA)
ATP6	648 (ATG/TAG)	559 (ATT/T)	652 (ATG/T)	246 (ATG/T)*	634 (ATG/T)	657 (ATG/TAA)	643 (ATG/T)	645 (TTG/TAG)	645 (ATG/TAA)	642 (TTG/TAA)	643 (ATG/T)	643 (ATG/T)	641 (ATA/TA)	643 (GTG/T)	645 (GTG/TAG)	642 (ATG/TAG)
ATP8	166 (ATA/T)	162 (ATG/TAG)	159 (GTG/TAG)	98 (CTT/TAG)*	168 (ATG/TAG)	123 (TTG/TAA)	153 (ATG/TAA)	147 (ATG/TAA)	138 (ATT/TAA)	153 (ATG/TAA)	186 (ATG/TAG)	151 (ATG/T)	151 (ATG/T)	157 (GTG/T)	159 (ATG/TAG)	153 (ATG/TAA)
N° tRNAs	22	22	22	22	22	22	22	25	24	22	22	22	22	22	22	22

The size of each genome, gene and the POR are in bp. Start and stop codons for protein-coding genes are indicated in parentheses.

* Partial sequences.

### Protein-coding Genes, Transfer RNAs, Ribosomal RNA Genes and Genome Organization

The size of the 13 protein-coding genes is similar to those described for other Eupulmonata species (Table 2), and their nucleotide composition reveals low GC content, where the lowest values were estimated for the NADH and ATP genes (Table S1). The most frequent start codon is ATG in six genes, followed by ATA in five genes and TTG in two genes (Table 2). On the other hand, five protein-coding genes (*COX1*, *COX3*, *NADH4*, *NADH5*, *NADH6*) use TAA as a stop codon, four (*NADH1*, *NADH2*, *NADH4L* and *ATP6*) use TAG, and other four (*COX2*, *Cytb*, *NADH3* and *ATP8*) use TXX (Table 2).

From the 22 annotated tRNAs 18 were identified with DOGMA [33], whereas the other four (*tRNA-S1*, *tRNA-I*, *tRNA-K* and *tRNA-W*) were identified with ARWEN v.1.2 [35] and tRNAscan-SE v.1.21 [36]. All tRNAs are spread over the entire genome, and are located on both strands (Fig. 2), and their length varies from 54 to 64 nucleotides. On the other hand, ribosomal RNAs showed similar characteristics to other Pulmonata rRNAs. Nevertheless, the large subunit (*16S-rRNA*) is shorter than those reported in other Pulmonata species (Table 2), and is encoded on the major strand, whereas the small subunit (*12S-rRNA*) is encoded on the minor strand (Fig. 2).

**Figure 3:**
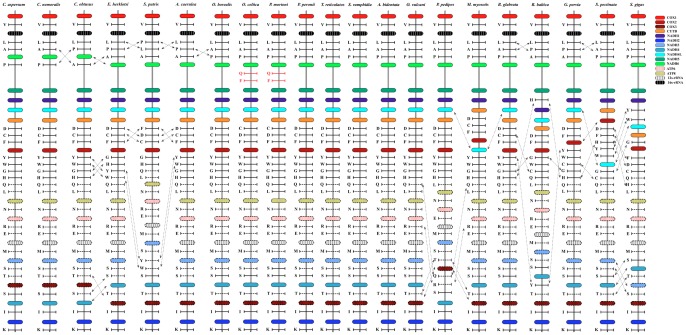
Linear representation of the gene order and t/rRNA locations in seven Pulmonata mollusc species. Mitochondrial genomes scaled to 100%. tRNAs are encoded in single letter code according to the amino acid they represent: A, Ala; G, Gly; P, Pro; T, Thr; V, Val; S, Ser; R, Arg; L, Leu; F, Phe; N, Asn; K, Lys; D, Asp; E, Glu; H, His; Q, Gln; I, Ile; M, Met; Y, Tyr; C, Cys; W, Trp). Boxes with shadow represent the reverse direction.

The gene order on the *C. aspersum* mitochondrial genome is the same as previously described for *Cepaea nemoralis* (Fig. 3). However, few differences were found when it was compared to *C. obtusus*, in which two tRNAs (*tRNA-A* and *tRNA-P*) are switched (Fig. 3). Similarly, when it was compared to *E. herklotsi*, *S. putris* and *A. caerulea* (i.e., Stylommatophora), some additional switches were detected in nine tRNAs (*tRNA-D*, *tRNA-C*, *tRNA-F*, *tRNA-Y*, *tRNA-W*, *tRNA-G*, *tRNA-H*, *tRNA-T*, *tRNA-S2*) and one protein-coding gene (*NADH* 4) (Fig. 3). Almost all of these differences are conserved in other Pulmonata species with the exception of *tRNA-P* that is inverted (Fig. 3).

### Intra-specific Divergence of *C. aspersum* mtDNA Genomes

**Figure 4:**
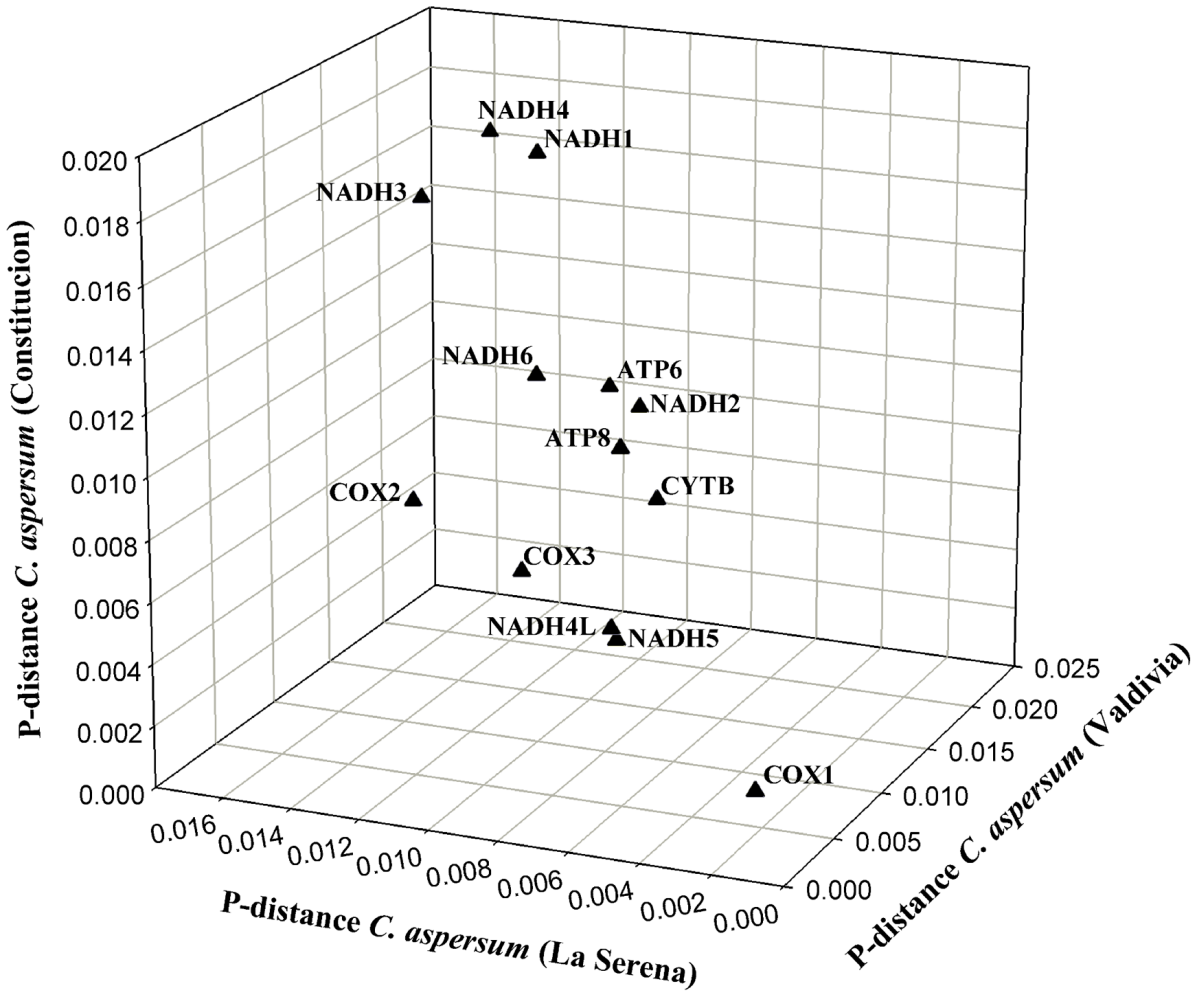
Intra-specific divergence of mitochondrial protein-coding genes in *Cornu aspersum*. P-distance corresponds to the number of base differences per site from averaging over all sequence pairs between genomes.

The intra-specific divergence analysis for each of the 13 protein-coding genes revealed that *COX1* has the lowest genetic differentiation among the three mitochondrial genomes (p-distance = 0.0019±4×10^−4^; Mean ± SD). The most divergent genes were *NADH1*, *NADH3* and *NADH4* with an average p-distance value of 0.0150. The other nine protein-coding genes showed intermediate values ranging from 0.0055 to 0.0120 (Fig. 4). When the large AT-rich intergenic spacer was included in the analysis, it almost doubled the p-distance values of most divergent protein-coding genes (i.e., p-distance = 0.025±2×10^−3^; Mean ± SD), showing high intra-specific genetic differentiation among populations.

### Phylogenetic Reconstruction

**Figure 5:**
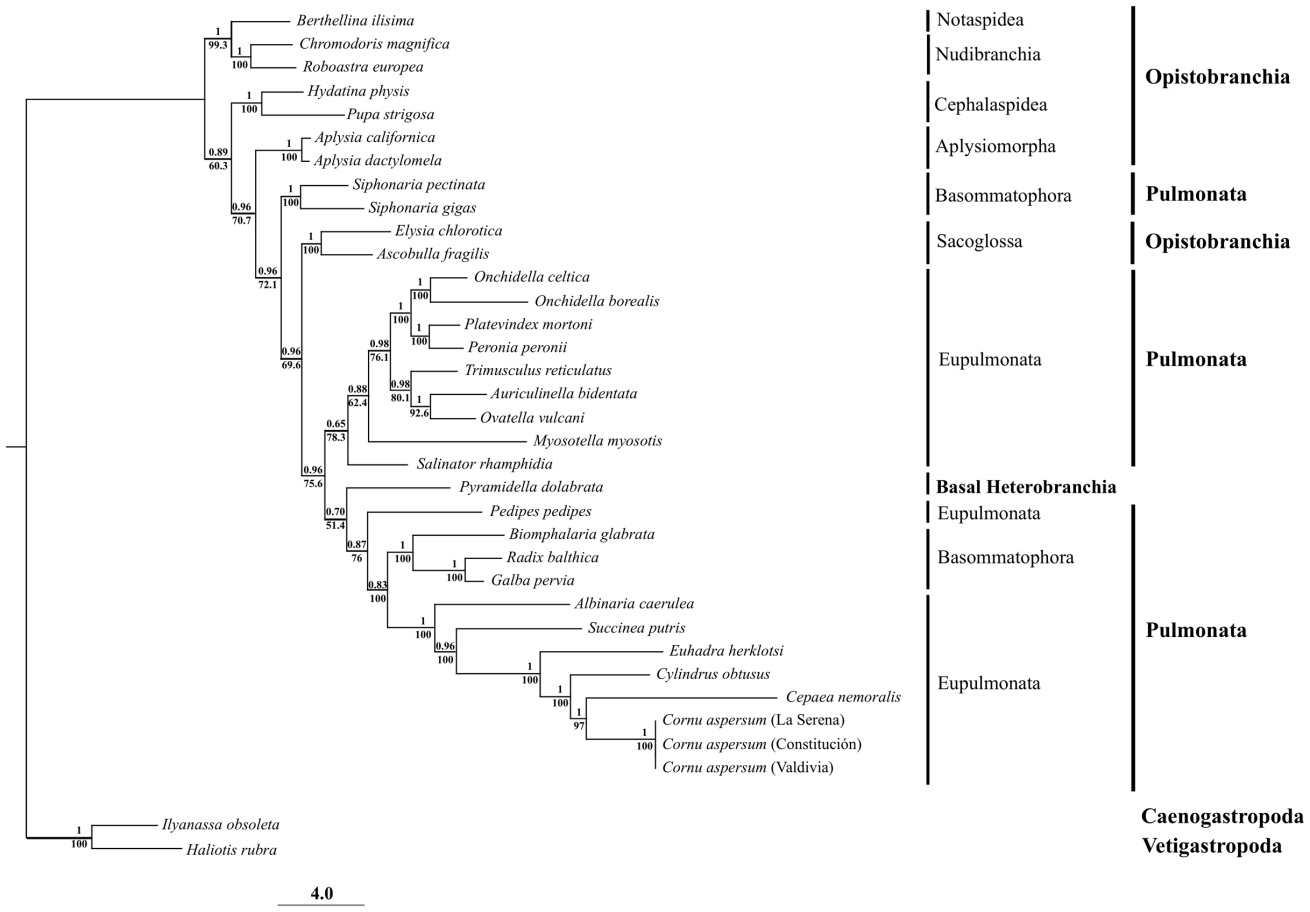
Maximum likelihood phylogram describing phylogenetic relationships among euthyneuran species (pulmonates and opisthobranchs). Numbers above the node correspond to Bayesian posterior probabilities, and those below the nodes to maximum likelihood bootstrap support. Sequences from *Ilyanassa obsoleta* and *Haliotis rubra* were the outgroup used to root the tree.

The Best Partition Scheme (BPS) for the concatenated alignment of the 13 protein-coding genes (12573 base positions) had 10 subsets partitions (Table S2). This BPS, and the estimated models of molecular evolution were used for both Bayesian and Maximum Likelihood analyses, which produced identical topologies, where most of the clades were strongly supported (Fig. 5). Phylogenetic reconstruction recovered Basommatophora as polyphyletic group, whereas Eupulmonata and Pulmonata as paraphyletic groups (Fig. 5). Bayesian and Maximum Likelihood analyses both recovered a clade containing the three *C. aspersum* genomes, with high support (Fig. 5), which in turn shares a most recent common ancestor with *Cepaea nemoralis*, followed by *Cylindrus obtusus* and *Euhadra herklotsi*, supporting the monophy of Helicidae (Fig. 5). Our analysis also supports the sister group relationship between Helicidae and the representative species of the family Succineidae (*Succinea putris*) and Clausiliidae (*Albinaria caerulea*), supporting the monophyly of the Stylommatophora clade (Fig. 5).

## Discussion

### General Features of the *C. aspersum* Mitochondrial Genome

In gastropods the length of the mitochondrial genomes usually vary from 13 to 17 kb (e.g., 13670 bp in *Biomphalaria glabrata,* 17575 bp in *Diodora aspera*), however, there are some exceptions such as the ribbed limpet *Lottia digitalis*, with a length of 26835 bp [43]. Here we found that the length of the mitochondrial genome of *C. aspersum* is similar to the other Eupulmonata species examined (Table 2). Although the mitochondrial genomes of gastropods are very compact [3], they contain intergenic non-coding regions of variable size that are responsible for differences in genome size [16]. In this regard, *C. aspersum*, *C. nemoralis* and *C. obtusus* have an AT-rich intergenic region, that contains the putative origin for mitochondrial DNA replication (POR), which is almost five times longer than other Eupulmonata species. This difference is probably due to insertions of tandem repeats in the POR region [44], which can be caused by gene rearrangements experienced by these species. Accordingly our results show that the POR region of *C. aspersum*, *C. nemoralis* and *C. obtusus* are located between *COX3* and *tRNA-S1* (i.e., First Serine), whereas in the other Pulmonata species is located between *COX3* and *tRNA-I* (Fig. 3).

The overall AT-content of the mitochondrial genome of *C. aspersum* is higher in comparison to *C. nemoralis* and *C. obtusus* (similar to that one observed in *E. herklotsi* and *A. caerulea*) but lower in comparison to *S. putris* (Table 2). Nevertheless, the average AT-content of the whole Stylommatophora clade is higher in comparison to other Eupulmonata orders (Table 2). The high AT-content is a common feature of most animal mitochondrial genomes [3], and it has been suggested that is the result of directional mutation pressure due to the lower energy requirement for opening the DNA strands with high AT content [45].

### Gene Order and Content

Given that the mitochondrial genome of pulmonate snails and slugs contain very little intergenic non-coding sequences and/or gene overlaps [11], [12], gene rearrangements are very rare considering that they would most likely disrupt some of the genes involved, rendering them non-functional. As a result of this, gene order in these species is well conserved [5]. It has been described that tRNA genes are more prone to switch their position than larger protein-coding and rRNA genes [5], [11], [16]. This pattern is observed in our study where *C. aspersum* has identical gene order and content to *C. nemoralis*, but both have small differences in comparison to the other stylommatophorans. These differences are explained by the inversions of some tRNA genes, and the *NADH4* gene (Fig. 3). Most of these inversions are maintained within Eupulmonata, but comparisons with Basommatophora revealed additional rearrangements that involve two additional tRNAs, and protein-coding genes (*COX2* and *NADH4L*) [4], [16].

Here we found that the size of the protein-coding, tRNA and rRNA genes is conserved within Eupulmonata (Table 2). The start and stop codon of the protein-coding genes in *C. aspersum* shows similarities to those of the other species analyzed. The presence of incomplete stop codons in *C. aspersum* seems to occur frequently in protein-coding genes of most of the mollusc mitochondrial genomes sequenced to date [4], [12], [16], [46], and it has been suggested that the transcripts of those genes would be modified to form a complete stop codon via post-transcriptional polyadenylation [47].

### Intra-specific Divergence of Mitochondrial Protein-coding Genes in *C. aspersum*

Intra-specific divergence analysis for each of the 13 protein-coding genes in *C. aspersum* revealed the existence of genes with high (*NADH1*, *NADH3* and *NADH4*) intermediate (*COX2*, *COX3*, *Cytb*, *NADH2*, *NADH4L*, *NADH5*, *NADH6*, *ATP6* and *ATP8*) and low (*COX1*) degrees of genetic differentiation (Fig. 4). In agreement with our results it has been shown that the *COX1* gene is one of the most conserved protein-coding genes in the mitochondrial genome of all metazoans [48], [49], while the most variable are the subunits of the NADH oxidoreductase and ATP synthase complexes [50]–[52]. The relatively low divergence in the mitochondrial genes that belong to the cytochrome c oxidase, the complex that catalyze the transfer of electrons from cytochrome c to oxygen, is most probably explained because this complex is the rate limiting step of the electron transport chain [53].

### Phylogenetic Analysis

The Bayesian and Maximum Likelihood phylogenetic tree topologies were identical, and clearly reject the hypothesis that Pulmonata is a monophyletic group (Fig. 5). This result is consistent with previous studies that place Pulmonata as a paraphyletic group within Euthyneura [4], [11], [16]. Additionally, our phylogenetic analysis recovered Basommatophora as a polyphyletic group, whereas Eupulmonata is a paraphyletic group (Fig. 5). The four species of onchidiids (*Onchidella celtica*, *O. borealis*, *Platevindex mortini* and *Peronia peronii*), were recovered as a highly supported monophyletic group (Fig. 5), sister to a clade containing *Trimusculus reticulatus* and two species of ellobiids (*Auriculinella bidentata* and *Ovatella vulcani*). The other two species of ellobiids (*Myostella myosotis* and *pedipes pedipes*) are recovered in two different clades within pulmonata (Fig. 5). Similarly to previous studies, *Pyramidella dolabrata* was found more closely related to pulmonates than to other Euthyneurans [4], [54]. On the other hand, the clade containing the newly sequenced *C. aspersum* share a most recent common ancestor with the banded snail *C. nemoralis* as expected given the current taxonomic classification [55], and also based on previous molecular studies [56], [57]. This clade was recovered as sister to *Cylindrus obtusus* and *Euhadra herklotsi*, supporting the monophy of Helicidae. Our analysis also supports the sister group relationship between Helicidae, and representative species from the family Succineidae and Clausiliidae, thus supporting the monophyly of Stylommatophora (land snails and slugs). This is in agreement with previous morphological [14], [55] and molecular studies [4], [11], [16].

### Conclusion

Pulmonata mitochondrial genomes display highly conserved structure and composition. However, with the sequencing of *C. aspersum* some general features of the mitochondrial genomes of Eupulmonata have been elucidated. Particularly, changes in gene order within Helicidae, due to the inversions of some tRNA genes and the *NADH4* gene, could be related to the longer size of the POR region in this clade compared to the other Eupulmonata species. The inclusion of the newly published sequence of the mitochondrial genome of the land snail *Cylindrus obtusus* [58] confirms the aforementioned characteristics for the family Helicidae, and together with *C. aspersum,* contribute to our understanding of the evolution of mtDNA within Stylommatophora and Pulmonata. Additionally, our phylogenetic results are consistent with the findings of Grande et al. [16] and White et al. [4] about the phylogenetic relationships among gastropods, and the monophyly of the lineage that clusters the land snails and slugs (i.e., Stylommatophora clade). Finally, our intraspecific comparisons revealed that *COX1* gene is one of the most conserved protein-coding genes in the mitochondrial genome of *C. aspersum*, while the most variable are the subunits of the NADH oxidoreductase complex.

## Supporting Information

Table S1 Compositional bias of the % GC content in the 13 protein-coding genes of the Pulmonata mitochondrial genomes used for the phylogenetic reconstruction.
(DOCX)

Table S2 Best Partition Scheme (BPS) and best-fit models of molecular evolution for the subsets partitions of the mitochondrial protein-coding genes alignment. The likelihood score (lnL) and the Bayesian Information Criterion (BIC) value were -123862 and 248843 respectively.
(DOCX)
