# Supplementary material for: The Complete Mitochondrial Genome of the Land Snail Cornu aspersum (Helicidae: Mollusca): Intra-Specific Divergence of Protein-Coding Genes and Phylogenetic Considerations within Euthyneura
Source: PLoS One. 2013 Jun 24;8(6):e67299. doi: 10.1371/journal.pone.0067299 (PMC3691120; doi:10.1371/journal.pone.0067299)
Supplement: Table S1 — Compositional bias of the % GC content in the 13 protein-coding genes of the Pulmonata mitochondrial genomes used for the phylogenetic reconstruction. (DOCX) [file pone.0067299.s001.docx]

Table S1. Compositional bias of the % GC content in the 13 protein-coding genes of the Pulmonata mitochondrial genomes used for the phylogenetic reconstruction.

| **Species** | **COX1** | **COX2** | **COX3** | **Cytb** | **NADH1** | **NADH2** | **NADH3** | **NADH4** | **NADH4L** | **NADH5** | **NADH6** | **ATP6** | **ATP8** |
| --- | --- | --- | --- | --- | --- | --- | --- | --- | --- | --- | --- | --- | --- |
| *Cornu aspersum* (La Serena) | 32.42 | 31.88 | 37.69 | 30.18 | 28.52 | 27.83 | 29.78 | 28 | 29.92 | 29.16 | 26.58 | 29.78 | 29.03 |
| *Cornu aspersum* (Constitución) | 32.29 | 32.61 | 37.31 | 30.18 | 28.52 | 27.94 | 29.51 | 27.92 | 29.55 | 29.1 | 26.58 | 30.09 | 29.03 |
| *Cornu aspersum* (Valdivia) | 32.35 | 31.88 | 37.18 | 30.44 | 28.64 | 28.15 | 30.33 | 28.23 | 29.92 | 29.16 | 26.99 | 29.78 | 29.03 |
| *Cepaea nemoralis* | 39.7 | 40.83 | 42.44 | 40.42 | 42.52 | 39.68 | 41.23 | 41.17 | 40.51 | 41.76 | 36.59 | 43.73 | 46.3 |
| *Cylindrus obtusus* | 39.69 | 40.47 | 42.44 | 39.11 | 37 | 36.61 | 41.18 | 37.02 | 32.93 | 36.73 | 35.74 | 43.47 | 45.91 |
| *Euhadra herklotsi* | 34.79 | 35.96 | 37.38 | 31.61 | 32.88 | 26.21 | 19.77 | 29.44 | 28.67 | 30.73 | 24.11 | 29.67 | 25.25 |
| *Albinaria caerulea* | 33.07 | 34.22 | 31.67 | 32.43 | 30 | 24.57 | 26.5 | 28.69 | 27.27 | 30.1 | 26.71 | 28.82 | 27.38 |
| *Succinea putris* | 30.1 | 28.7 | 27.97 | 26.38 | 23.39 | 16.31 | 20.8 | 20.44 | 20.15 | 21.37 | 16.11 | 23.14 | 16.26 |
| *Onchidella celtica* | 38.9 | 40.97 | 43.5 | 41 | 41.39 | 42.24 | 40.17 | 39.3 | 38.95 | 40.46 | 41.94 | 44.81 | 39.46 |
| *Onchidella borealis* | 35.17 | 36.46 | 37.07 | 34.15 | 34.99 | 32.68 | 32.76 | 32.41 | 30.77 | 33.46 | 30.94 | 34.74 | 25.49 |
| *Platevindex mortoni* | 37.55 | 39.94 | 39.63 | 37.16 | 35.94 | 37.68 | 31.7 | 36.05 | 32.64 | 36 | 38.89 | 36.43 | 30.43 |
| *Peronia peronii* | 35.89 | 37.84 | 41.31 | 36.22 | 36.2 | 36.95 | 34.56 | 36.45 | 33.33 | 35.01 | 35.68 | 35.05 | 31.37 |
| *Trimusculus reticulatus* | 39.36 | 41.44 | 39.64 | 39.1 | 39.18 | 37.49 | 39.5 | 38.85 | 37.19 | 38.45 | 38.6 | 38.16 | 37.63 |
| *Salinator rhamphidia* | 38.9 | 39.37 | 41.54 | 40.09 | 39.29 | 35.71 | 38.79 | 38.29 | 38.71 | 37.16 | 33.97 | 38.32 | 36.67 |
| *Myosotella myosotis* | 42.89 | 48.28 | 46.46 | 46.22 | 46.15 | 44.73 | 45.35 | 43.98 | 45.36 | 45.35 | 50.85 | 46.17 | 47.33 |
| *Auriculinella bidentata* | 42.27 | 45.56 | 44.02 | 45.5 | 40.4 | 42.33 | 38.98 | 43.1 | 44.65 | 44.2 | 44.1 | 45.17 | 40.38 |
| *Ovatella vulcani* | 42.89 | 48.28 | 46.46 | 46.22 | 46.15 | 44.73 | 45.35 | 43.98 | 45.36 | 45.35 | 50.85 | 46.17 | 47.33 |
| *Pedipes pedipes* | 39.69 | 39.35 | 41.92 | 38.39 | 36.77 | 38.08 | 38.71 | 38.86 | 35.07 | 37.57 | 35.06 | 41.59 | 34.64 |
| *Biomphalaria glabrata* | 30.19 | 31.17 | 28.04 | 28.97 | 27.38 | 20.86 | 18.68 | 23.86 | 25.67 | 23.44 | 19.26 | 25.98 | 20.95 |
| *Radix balthica* | 33.2 | 31.7 | 34.1 | 31.8 | 30.57 | 25.94 | 26.1 | 27.82 | 25.99 | 28.75 | 21.91 | 28.79 | 38.99 |
| *Galba pervia* | 33.07 | 30.33 | 29.95 | 30.6 | 27.06 | 23.76 | 25.96 | 25.04 | 24.73 | 25.59 | 23.58 | 25.33 | 19.05 |
| *Siphonaria pectinata* | 33.27 | 33.18 | 36.29 | 34.68 | 32.88 | 32.05 | 33.05 | 32.58 | 30.85 | 31.05 | 31.37 | 30.77 | 27.21 |
| *Siphonaria gigas* | 39.48 | 40.92 | 41.23 | 40.18 | 39.09 | 39.07 | 38.89 | 36.51 | 40.48 | 37.81 | 36.2 | 39.42 | 33.33 |
