# Supplementary material for: The Complete Mitochondrial Genome of the Land Snail Cornu aspersum (Helicidae: Mollusca): Intra-Specific Divergence of Protein-Coding Genes and Phylogenetic Considerations within Euthyneura
Source: PLoS One. 2013 Jun 24;8(6):e67299. doi: 10.1371/journal.pone.0067299 (PMC3691120; doi:10.1371/journal.pone.0067299)
Supplement: Table S2 — Best Partition Scheme (BPS) and best-fit models of molecular evolution for the subsets partitions of the mitochondrial protein-coding genes alignment. The likelihood score (lnL) and the Bayesian Information Criterion (BIC) value were -123862 and 248843 respectively. (DOCX) [file pone.0067299.s002.docx]

Table S2. Best Partition Scheme (BPS) and best-fit models of molecular evolution for the subsets partitions of the mitochondrial protein-coding genes alignment. The likelihood score (lnL) and the Bayesian Information Criterion (BIC) value were -123862 and 248843 respectively.

| **Subset** | **Partition for codon position** | **Model** |
| --- | --- | --- |
| 1 | Cox1_1, Cox2_1, Cox3_1, Cytb_1, Nadh1_1 | GTR+I+G |
| 2 | Cox1_2, Cox2_2, Cox3_2, Cytb_2 | GTR+G |
| 3 | Cox1_3, Cox2_3, Cytb_3 | HKY+G |
| 4 | ATP6_3, ATP8_3, Cox3_3, Nadh3_3 | HKY+G |
| 5 | ATP6_2, Nadh1_2, Nadh3_2, Nadh4_2, Nadh5_2 | GTR+I+G |
| 6 | Nadh1_3, Nadh4_3, Nadh5_3 | HKY+G |
| 7 | ATP6_1, Nadh2_1, Nadh3_1, Nadh4L_1, Nadh4_1, Nadh5_1, Nadh6_1 | GTR+I+G |
| 8 | Nahd2_2, Nadh4L_2, Nadh6_2 | GTR+G |
| 9 | Nahd2_3, Nadh4L_3, Nadh6_3 | HKY+G |
| 10 | ATP8_1, ATP8_2 | F81+G |
